# Pregnancy and Diabetes

**Published:** 1910-09-17

**Authors:** 


### PREGNANCY AND DIABETES.

The occurrence of reducing substances in the urine of pregnant women is not uncommon, but very often the reducing substance is lactose and not glucose. Occasionally, however, actual glycosuria does occur in association with pregnancy, and the question arises as to what the prognosis is and how the patient should be dealt with. The cases may be grouped into two main classes—namely, first, pregnant women who pass sugar in their urine when their diet contains a full amount of sugar or starch, but not when these articles are even slightly limited, and, secondly, those suffering from true and persistent diabetic glycosuria. It is very probable that these two groups of cases merely merge into one another without any actual distinction in kind; the difference in degree, however, is so marked that they need to be put into separate categories for clinical purposes. The discovery of the sugar in the urine in the first class is generally accidental, but in the second there are usually symptoms affecting either the nervous system or the body generally. As a matter of fact, the occurrence of any important amount of fermentable sugar in the urine of pregnant women is remarkably rare, and it is still rarer for the combined conditions to prove fatal. The age period at which diabetes is most fatal is between twenty and forty, and this is just the age when pregnancy is apt to occur, so that most of the cases of fatal diabetes in pregnant women might readily have proved fatal had the diabetes occurred without pregnancy coinciding with it. Nevertheless, there are a certain number of cases of pregnancy in which acidosis and the symptoms associated with the latter occur, and lead to symptoms of poisoning of which one of the best examples perhaps is the intractable vomiting of pregnancy; the serious symptoms of diabetes are also due to acidosis, that is to say, to the effects of an abnormal kind of metabolism which leads to the production of acid substances, especially oxybutyric acid, diacetic acid, and acetone. If both pregnancy by itself and also diabetes tend to cause acidosis there must be cases when these tendencies coincide and lead to graver results than either would separately. Hence it is clear that if one were confronted with the question as to whether an unmarried girl suffering from diabetes should get married, the advice should most emphatically be no. If a woman is already married when diabetes sets in, conception should certainly be avoided if possible. Should a diabetic woman become pregnant, there is no immediate necessity for terminating the pregnancy if no symptoms or signs of serious acidosis exist; but the treatment should be such as to limit the tendency to acidosis to the very greatest extent possible, and to this end it is most important that the best dietary for the case should be ascertained as soon as possible, the maximum amount of carbo-hydrate being allowed, the patient being permitted plenty of exercise short of fatigue, and she should in all respects live as healthy an open-air life as may be. It is a great mistake in these cases to rely upon the total amount of sugar in the urine as a guide as to whether the patient is better or worse. A strict reduction in the amount of carbo-hydrate in the dietary may serve to reduce the sugar in the urine very considerably, but it may at the very same time greatly increase the amount of acetone and diacetic acid. The patient is often in less danger of coma from acidosis with a larger amount of sugar being passed than with a smaller, and far more important than the sugar is the amount of acetone present, the best measure of the degree of acidosis being the estimation of the amount of ammonia in the urine. The less the ammonia the less the acidosis, and the less the danger to the patient.

